# A mixed-methods assessment of disclosure of HIV status among expert mothers living with HIV in rural Nigeria

**DOI:** 10.1371/journal.pone.0232423

**Published:** 2020-04-30

**Authors:** Angela Odiachi, Nadia A. Sam-Agudu, Salome Erekaha, Christopher Isah, Habib O. Ramadhani, Homsuk E. Swomen, Manhattan Charurat, Llewellyn J. Cornelius

**Affiliations:** 1 Research Consultant, Abuja, Nigeria; 2 International Research Center of Excellence, Institute of Human Virology Nigeria, Abuja, Nigeria; 3 Division of Epidemiology and Prevention, Institute of Human Virology, University of Maryland School of Medicine, Baltimore, Maryland, United States of America; 4 Sexual, Reproductive Health and Gender Unit, United Nations Population Fund, Abuja, Nigeria; 5 School of Social Work and College of Public Health, University of Georgia Athens, Athens, Georgia, United States of America; Yale University Yale School of Public Health, UNITED STATES

## Abstract

**Background:**

Peer support provided by experienced and/or trained “expert” women living with HIV has been adopted by prevention of mother-to-child transmission of HIV (PMTCT) programs across sub-Saharan Africa. While there is ample data on HIV status disclosure among non-expert women, there is little data on disclosure among such expert women, who support other women living with HIV.

**Objective:**

This study compared HIV disclosure rates between expert and non-expert mothers living with HIV, and contextualized quantitative findings with qualitative data from expert women.

**Methods:**

We compared survey data on HIV disclosure to male partners and family/friends from 37 expert and 100 non-expert mothers living with HIV in rural North-Central Nigeria. Four focus group discussions with expert mothers provided further context on disclosure to male partners, extended family and peers. Chi square and Fisher’s exact tests were applied to quantitative data. Qualitative data were manually analyzed using a Grounded Theory approach.

**Results:**

Two-thirds of the 137 participants were 21–30 years old; 89.8% were married, and 52.3% had secondary-level education. Disclosure to male partners was higher among expert (100.0%) versus non-expert mothers (85.0%), p = 0.035. Disclosure to anyone (93.1% vs 80.8%, p = 0.156), and knowledge of male partners’ HIV status were similar (75.7% versus 66.7%, p = 0.324) between expert and non-expert mothers, respectively. With respect to male partners, HIV serodiscordance rates were also similar (46.4% vs 55.6%, p = 0.433). Group discussions indicated that expert mothers did not consistently disclose to their mentored clients, with community-level stigma and discrimination stated as major reasons for this non-disclosure.

**Conclusions:**

Expert mothers experience similar disclosure barriers as their non-expert peers, especially regarding disclosure outside of intimate relationships. Thus, attention to expert mothers’ coping skills and disclosure status, particularly to mentored clients is important to maximize the impact of peer support in PMTCT.

**Clinical trials registration:**

Clinicaltrials.gov registration number NCT 01936753 (retrospective), September 3, 2013.

## Introduction

Prevention of mother-to-child transmission of HIV (PMTCT) programs increasingly engage lay women living with HIV (WLHIV) to deliver services and facilitate optimal PMTCT outcomes for their peers [1–8]. These exemplary WLHIV, often called expert mothers, have successfully completed the PMTCT cascade at least once, ideally are virally suppressed, and have an HIV-negative child, indicating successful PMTCT outcomes [4]. The PMTCT cascade is a series of services required for optimal maternal-infant benefits. These steps include maternal access to antenatal care, HIV test, and antiretroviral therapy (ART); and infant prophylaxis, early HIV diagnosis, and final confirmation of HIV status. Expert mothers serve as model service users, and are presented as such for their peers to emulate. They are charged with behaviorally influencing their peers to complete the PMTCT cascade, through counselling and support for uptake of services, retention in care, drug adherence, psychosocial well-being, and HIV status disclosure to male partners [8–10].

Maternal HIV status disclosure to male partners is an important facilitator of optimal outcomes for mother-infant pairs in the PMTCT cascade. Studies in high HIV-burden countries demonstrate that disclosure to male partners is associated with better service utilization, drug adherence, exclusive breastfeeding, and facility delivery rates [11–13]. Non-disclosure to male partners is a barrier to women’s (and men’s) participation in PMTCT, and poses higher risks for maternal-to-child HIV transmission [14–17]. Ultimately, the potential to achieve the desired PMTCT outcomes of HIV-free infant survival and sustained maternal viral suppression is limited by maternal non-disclosure to male partners.

The engagement of expert mothers to effect behavioral change among PMTCT clients is promising for high HIV-burden settings such as Nigeria. Nearly 200,000 WLHIV in Nigeria are pregnant every year, with only 30% of them accessing ART [18, 19]. This gap increases mother-to-child transmission of HIV risk, and contributes to an estimated 36,000 new child HIV infections every year [19]. The sub-optimal maternal ART coverage and high mother-to-child transmission rates are likely worse in rural areas, where maternal healthcare utilization is poor. For example, rural Nigeria has lower skilled antenatal care uptake (46.5%) than urban areas (86.0%); and facility delivery occurs for only 21.9% of rural, versus 61.7% of urban women [20]. Clearly, it is prudent to generate evidence for both biomedical and behavioral PMTCT interventions in Nigeria, especially in rural areas where gaps may be highest.

There is ample data on the impact of expert mothers and similar peer support on PMTCT outcomes, disclosure rates, and experiences among WLHIV in general [2–7, 21–25]. However, there is little data on disclosure among expert mothers functioning as mentors for other WLHIV. In this study, we assessed HIV status disclosure rates to male partners and others among expert mothers, in comparison to non-expert mothers in rural North-Central Nigeria. Secondly, we explored disclosure experiences among expert mothers to explain and contextualize findings on their comparative disclosure rates.

## Materials and methods

### Study setting

This study was conducted between December 2012 and November 2013 at 16 primary healthcare centers and four referral facilities (three secondary, one tertiary) in rural North-Central Nigeria. These rural communities were the study communities for MoMent (Mother Mentor) Nigeria, a PMTCT implementation research study investigating the impact of structured versus unstructured peer support on PMTCT outcomes [26]. Expert mother support, albeit unstructured, was provided for pregnant and postpartum WLHIV as part of routine PMTCT care at all facilities in the study communities. The data reported in this paper were collected during the formative component of the MoMent Nigeria study.

### Study design, participant recruitment and data collection

This concurrent mixed methods study combined quantitative data from surveys with qualitative data from focus group discussions (FGDs) (Table 1).

**Table 1:** Mixed-methods study design among women living with HIV.

Study Component and Data Collected	Expert mothers^a^ N = 37	Non-expert mothers^b^ N = 100
**Quantitative surveys**		
• Sociodemographic data		
• HIV disclosure information	Yes	Yes
• Knowledge of male partner HIV status		
**Qualitative studies (Focus Groups)**	Yes	No
**Preset code words**		
• Disclosure, stigma, discrimination, support		

^a^ Women providing one-on-one peer support for ≥1 year and/or attending mother-to-mother support groups for ≥3 months

^b^ Women with no history or experience in mentoring peers with no, or <3 months support group exposure

#### Study participants

All participants were WLHIV ≥18 years old, selected by typical case purposive sampling. Participants were recruited in two groups (Table 1); first, expert mothers, defined as WLHIV who had been providing one-on-one peer support in MoMent study communities and/or attended monthly facility-based mother-to-mother peer support groups for WLHIV. For the purpose of this study, eligible expert mothers were to have served as peer counsellor for ≥1 year or attended peer support groups for ≥3 months. Given the rich PMTCT education and social empowerment they provide, these support groups are very often the source for recruiting expert mothers to provide one-on-one peer support in PMTCT programs; members often rotate in their tenure as peer counsellors. Thus, members of these groups were designated expert mothers in this study. Non-expert mothers were defined as WLHIV accessing care at facilities in study communities, who had no history or experience in providing peer support, and/or had less than three months of attendance at monthly mother-to-mother support groups.

#### Recruitment

In our study setting, an average of two expert mothers are assigned to each health facility and typically provide services to between 15 and 25 clients. Women were recruited from study healthcare facilities by clinic staff. After indicating interest, potential participants were asked to present for the short survey and FGD at the study facility or other pre-determined venue on the scheduled. For the survey among non-expert mothers, the aim was to recruit five women from each of the selected 20 facilities located in MoMent’s study communities, for a target sample size of 100. This was done in order to adequately represent each study community as well as pregnant and postpartum WLHIV. The surveys and FGDs were conducted on non-clinic days in private rooms at the study facilities or at private venues away from the clinic.

#### Quantitative data collection

The survey document was pre-tested among 10 women and revised before survey data were collected. Sociodemographic and disclosure data were collected individually and privately for all participants. Each interviewer-administered survey lasted 15 to 30 minutes, and obtained information on disclosure to family, friends and male partners (see Supporting Information for survey).

#### Qualitative data collection

The aim was to conduct multiple focus groups among expert mothers, collecting and analyzing data via an iterative process until thematic saturation was achieved. The FGD guide was developed in English (see Supporting Information for guide), but the discussions were conducted in English, Pidgin English (a local variant of English) and Hausa (the dominant local language) by trained, multilingual staff, including authors NASA, SE, and LJC. Each FGD was audio-recorded and lasted 1 ½ to 2 hours. Clinic staff at the study sites neither participated in, nor observed the FGDs. Two trained facilitators moderated each FGD using the guide, and an observer recorded non-verbal cues as part of field notes. Ultimately, four FGDs were conducted with expert mothers—two FGDs each for mentor mothers and mother-to-mother support group members, with eight to 10 women in each focus group.

### Data analyses

#### Quantitative analysis

Participant socio-demographic and disclosure data were subjected to descriptive analyses. Expert and non-expert mother disclosure data were subsequently compared by Chi-square and Fisher’s exact tests, as applicable, using Statistical Package for Social Sciences (SPSS) version 16.0 for Windows. Statistical significance was set at p < 0.05.

#### Qualitative analysis

The audio-taped FGDs were transcribed verbatim (and translated, where necessary) in English by study staff who facilitated the FGDs. Back-translation was not formally conducted, but transcripts were reviewed by co-facilitators or co-observers for consistency. Using the constant comparative approach of classic Grounded Theory [27] transcripts were manually coded, organized, and analyzed, by general themes (from the FGD guide) and emerging themes from the data. Grounded Theory was our theoretical framework of choice because we aimed to capture a broad range of expert mother disclosure experiences without any preconceived notions or theories about these experiences, for which there has been limited prior evidence. We organized our findings using the consolidated criteria for reporting qualitative research (COREQ) checklist [28] (see Supporting Information).

#### Ethical approval

The study was approved by the Nigeria National Health Research Ethics Committee, the Ethics Review Committee of the World Health Organization, and the Institutional Review Boards of the University of Maryland Baltimore and the University of Georgia Athens. All FGD and survey participants provided written informed consent prior to group discussions and survey completion.

## Results

A total of 137 eligible WLHIV were recruited: 37 expert (18 mentor mothers and 19 support group mothers) and 100 non-expert mothers. Expert mothers providing one-on-one peer support had been serving as mentor mothers for a median of 22 months (IQR 12–24 months), while expert mothers in support groups had been attending the groups for a median of 10 months (IQR 3–24 months). The majority (~70%) of our study cohort was 21–30 years (Table 2). About half (52.3%) had attained at least secondary-level education. However, non-expert mothers were more likely to have obtained secondary-level or higher education than expert mothers (61.6% versus 20.6%, p = 0.001). Nearly 90% (123/137) of participants were married, with 3.3% (4/123) married women in polygamous unions. Expert mothers were more likely to be married compared to non-expert mothers (100% versus 86%, p = 0.012).

**Table 2:** Socio-demographic characteristics of women living with HIV.

Characteristics	Expert mothers N = 37^a^ n (%)	Non-expert mothers N = 100^b^ n (%)	Total N = 137 n (%)	*P* value^c^
**Age (years)**	N = 37	N = 100	N = 137	
< 21	1 (2.7)	7 (7.0)	8 (5.8)	0.478
21–30	24 (64.9)	69 (69.0)	93 (67.9)	
31–40	12 (32.4)	24 (24.0)	36 (26.3)	
**Educational attainment**	N = 37	N = 99	N = 136	
None	7 (24.2)	11 (11.1)	18 (14.1)	**0.001**
Primary	16 (55.2)	27 (27.3)	43 (33.6)	
Secondary	3 (10.3)	47 (47.5)	50 (39.1)	
Tertiary and beyond	3 (10.3)	14 (14.1)	17 (13.2)	
**Religion**	N = 37	N = 98	N = 135	
Christian	23 (62.2)	75 (76.5)	98 (72.6)	0.129
Muslim	14 (37.8)	23 (23.5)	37 (27.4)	
**Marital status**	N = 37	N = 100	N = 137	
Single^d^	0 (0.0)	14 (14.0)	14 (10.2)	**0.012**
Married	37 (100.0)	86 (86.0)	123 (89.8)^e^	
**Number of living children**	N = 33	N = 98	N = 131	
None	1 (3.0)	19 (19.5)	20 (15.3)	0.082
1–2	16 (48.5)	46 (46.9)	62 (47.3)	
3–4	12 (36.4)	26 (26.5)	38 (29.0)	
≥ 5	4 (12.1)	7 (7.1)	11 (8.4)	

^a^Includes 18 mentor mothers and 19 support group mothers

^b^Includes 51 pregnant women

^c^Fisher’s Exact test

^d^Includes single, divorced, separated and widowed women

^e^Includes 4 women in polygamous unions

### Results from quantitative data analysis

#### Disclosure rates among expert and non-expert mothers

Disclosure to anyone (male partner, family member or friend) was similar between expert and non-expert mothers (93.1% versus 80.8%, p = 0.156). Notably, two expert mothers had never disclosed to anyone (Table 3).

**Table 3:** Rates of HIV status disclosure by expert and non-expert women living with HIV.

	Expert mothers N = 37 n (%)	Non-expert mothers N = 100 n (%)	*P* value^a^
**Disclosed to anyone**^b^			
Yes	27 (93.1)	80 (80.8)	0.156
No	2 (6.9)	19 (19.2)	
Missing data/no response	8	1	
**Disclosed to male partner**^c^	**N = 27 n (%)**	**N = 80 n (%)**	
Yes	27 (100.0)	68 (85.0)	**0.035**
No	0 (0.0)	12 (15.0)	
Missing data/no response	0	0	

^**a**^Fisher’s exact test

^b^Includes male partners, friends and family members

^c^Denominator limited to women who have disclosed to anyone

For expert mothers who reported disclosure to anyone, disclosure was to only male partners. In other words, per self-report, no expert mother had disclosed to family members nor friends. Male partner disclosure was significantly higher among expert compared to non-expert mothers (100.0% versus 85.0%, p = 0.035).

#### Knowledge of male partner HIV status

Expert mothers were no more likely than non-expert mothers to know their male partners’ HIV status (75.7% versus 66.7%, p = 0.324), (Table 4). Reported HIV serodiscordance was also similar for expert and non-expert mothers (46.4% versus 55.6%, p = 0.433).

**Table 4:** Knowledge of male partner HIV status among expert and non-expert women living with HIV.

Knowledge of male partner HIV status	Expert mothers N = 37 n (%)	Non-expert mothers N = 100 n (%)	*P* value^a^
Known	28 (75.7)	54 (66.7)	0.324
Unknown	9 (24.3)	27 (33.3)	
Missing data/no response	0	19	
**Male partner HIV status**^b^	**N = 28 n (%)**	**N = 54 n (%)**	
Positive	15 (53.6)	24 (44.4)	0.433
Negative	13 (46.4)	30 (55.6)	

^a^Chi square test

^b^Denominator limited to known male partner status

### Results from qualitative data analysis

We sought to understand and explain the quantitative disclosure findings among expert mothers with narratives from FGDs among this group.

**Fig 1:**
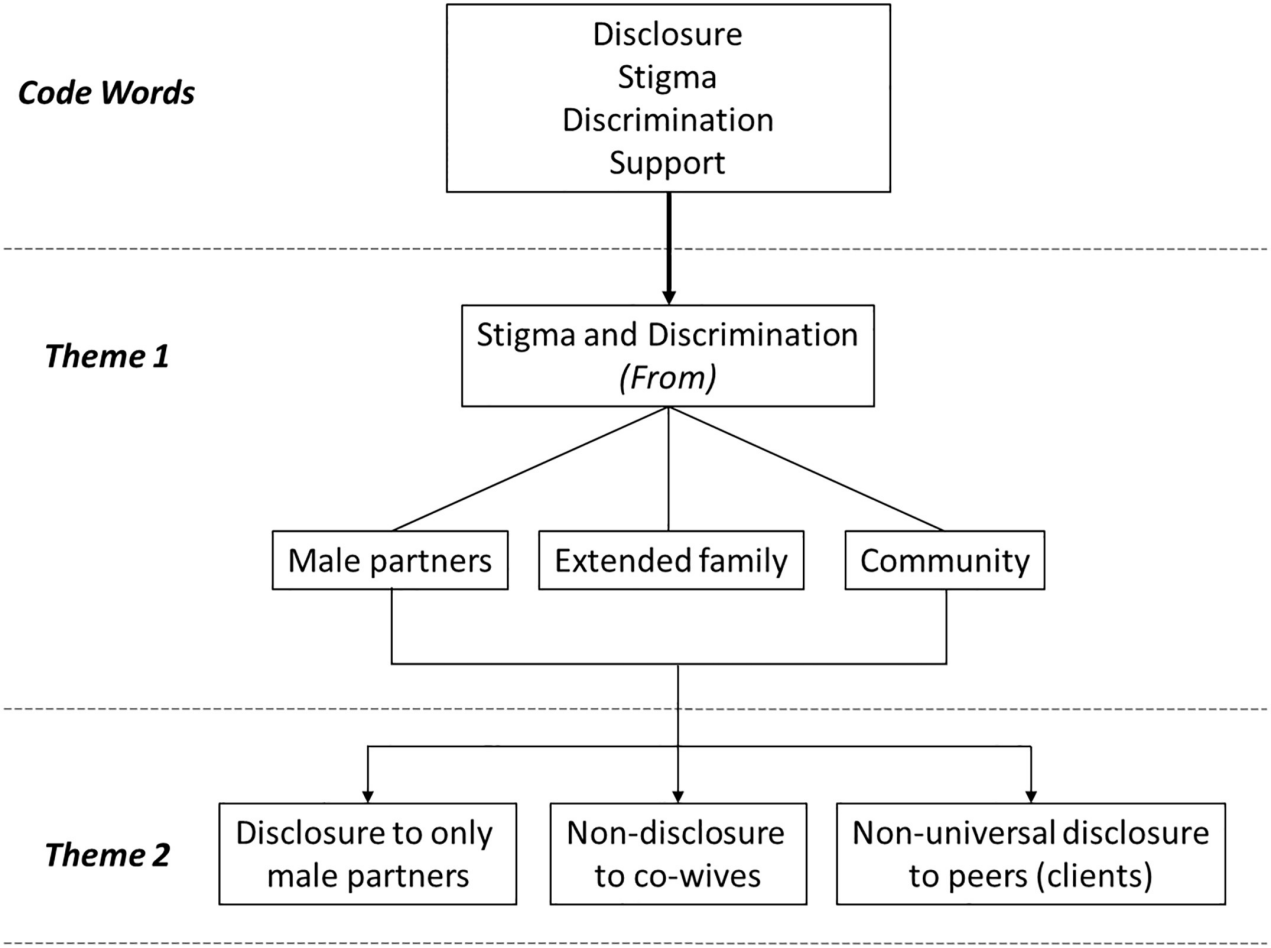
Themes from qualitative data analysis: Disclosure among expert mothers.

Expert mothers reported personally experiencing and/or witnessing client stigma and discrimination from the community, extended family, and male partners. This contributed to varying degrees of disclosure (rather than the expected universal disclosure) by expert mothers to these groups (Fig 1).

#### Stigma and discrimination from the extended family and community

Even as expert mothers were empowered to support peers in their homes and the community, community-level stigma and discrimination against them and their clients made it difficult to disclose and identify as WLHIV.

‘*One of my clients said that she is selling food*. *Somebody came to clinic and saw her carrying her folder*. *The person said*, “*I am not going to eat your food anymore*.”

(Mentor mother 1, FGD1).

‘*In the* [client’s] *family house*, *there is one particular cup that they usually hide*. *It is one of them that told us that when we* [mentor mothers] *visit*, *they go and hide that cup because they don*’*t want us to use that cup*’

(Mentor mother 7, FGD2).

However, some expert mothers emphatically stated that they had personally largely overcome stigma, and portrayed less apprehension regarding community members’ opinions or reactions to their HIV-positive status.

‘*I can say that as far as you present yourself as a mentor mother*, *you need to overcome the stigma*. *That is the reason why we are role models*. *For me*, *I have overcome stigma*’

(Mentor mother 1, FGD1).

‘*We faced that* [stigma], *but it was more previously*. *But now it is just like it*’*s the order of the day*. *Most people have it* [HIV] *so it*’*s not a big deal any more*, *especially in my own community*’

(Mentor mother 5, FGD2).

#### Stigma and discrimination from male partners

For some expert mothers, working as a mentor mother or participating in support group meetings resulted in reduced financial and other support from male partners. This was presumably because these expert mothers had secured employment and/or empowerment, and were therefore considered by male partners to not need extra support. Additionally, some expert mothers experienced neglect and separation from their male partners after disclosure. All of these experiences were shared in the context of universal male partner disclosure-for women whose quantitative data were available (Table 3).

‘*Since I started this mentor mother work*, *my husband has stopped supporting me with little things that I need*, *like soap and small household items*…*And during our support group meetings*, *other women usually complain that their husbands no longer provide for them*’

(Mentor mother 6, FGD2).

‘*My husband is negative*, *I am positive*, *so I told him*. *This thing* [HIV] *was somehow bringing problems to me*. *He divorced me the first time*. *He then divorced me a second time*, *saying that if he stayed with me*, *he will contract the disease [HIV]*’

(Mentor mother 8, FGD1).

Some expert mothers discussed neglect, sex denial or blame from male partners even when these partners were also HIV-positive.

‘*Some* [clients] *will say that because of their HIV status*, *their husbands are no more sleeping with them*. *So when the woman needs a man and that man cannot give what that woman wants*, *that is the worst stigma*. *Another stigma is that some men*, *when they come to clinic after counselling*, *even if they came out positive*, *their wife came out positive*, *they will still be throwing the blame on the woman*’

(Mentor mother 1, FGD1).

However, expert mothers identified male partners (versus extended family or community members) as still the best option for disclosure due to the intimate nature of these relationships, the opportunity for sexual partners to test for HIV, and the couple’s mutual need for confidentiality regarding their HIV status.

‘*As for the environment*, *you don*’*t go about discussing your problem* [HIV] *with people around*, *except your husband*. *Even your mother*, *your sisters*, *your brothers might not know*. *The reason why you should tell him* [husband] *is because he is the only person that you are being intimate with*, *for him to go and see if he is free* [of HIV] *or not*, *then that will help you know where you stand*. *Apart from these two people -nurses here and your spouse*—*I don*’*t think you will feel free discussing your problem with people*’

(Support group mother 2, FGD3).

#### Non-disclosure of HIV status to co-wives in polygamous unions

Some expert mothers in polygamous marriages admitted that their co-wife/wives did not know about their HIV-positive status. In most of these cases, only the husband knew.

‘*You can*’*t tell the other woman* [co-wife]. *You only keep quiet*. *How am I sure that if I tell her that I am positive*, *she will be able to take me the way I am*, *the way we related before*? *It*’*s only if you know your [co-wife] well*, *how intimate you people are*, *that*’*s when you will expose your status to her*’

(Mentor mother 1, FGD2).

Mentor mothers also described similar experiences among clients who were in polygamous unions.

‘*I know of a couple*, *nobody knows the status of the first wife*. *But the second wife and the husband collect their HIV drugs from here* [clinic], *leaving the other one*. *I*’*ve tried to talk to the woman and she said the husband said she should not tell her* [first wife], *because if she does*, *the first wife will go and expose the information*’

(Mentor mother 5, FGD2).

‘*We have a client like that*. *The man was positive and he refused to tell his wife*…*until he got married to another woman*. *The new wife came here* [clinic] *for ante-natal and discovered she was HIV-positive*. *She said the drugs they gave her are the kind she has been giving her husband*. *I told her to ask her husband the reason he is taking these drugs regularly*. *It was much later that he told her that he was positive*’

(Mentor mother 4, FGD2).

#### Expert mother disclosure to peers

Expert mothers discussed how disclosure to peers was more appealing because of the shared experience of living with HIV with other women.

‘*If I am to be a mentor mother to somebody whom I know is suffering the same thing I am suffering* [HIV], *I don*’*t think I will hold back from telling the person anything because if the person goes out to talk about it*, *the person is equally talking about herself*. *But if you*’*re not* [HIV-positive], *I doubt I will tell you*’

(Support group mother 2, FGD3).

Despite the expressed willingness and preference for peer disclosure in FGDs, our survey data indicated that a few mentor mothers had not disclosed to anyone at all, suggesting that they had not even disclosed their HIV-positive status to their clients.

‘*The one mentor mother that was assigned to me before*, *she did not disclose her status to me and I did not know she was hiding it*. *It was the day we came for a* [support group] *meeting here*, *after about a year plus*, *that I came to know*. *Since then she doesn*’*t come to my house anymore*; *at most she will call on phone*’

(Support group mother 3, FGD3).

‘*There is this one mentor mother who visits me*…*But I found out* [she was HIV-positive]…S*he did not tell me herself*. *She has been hiding it* [HIV-positive status]’

(Support group mother 4, FGD3).

The shroud of secrecy around HIV status and disclosure was further reinforced in mentor-mother-client interactions during home visits. Mentor mothers and their clients would talk in “code” in order to conceal the client’s—and perhaps the mentor mother’s—HIV status.

‘*The person* [mentor mother] *that was attached to me told me that everything has a name*. *If she wanted to remind me of my drugs she will just say the code and I know she is reminding me of my drugs*. *She might just tell me* “*remember our meeting o*.” *I know I am not going for any meeting*; *I know that the* “*meeting*” *is time for my drugs*. *So we always have names for some things and anybody else will be confused* … *So you must not expose yourselves*.’

(Support group mother 2, FGD3).

## Discussion

Our study provides some insight into comparative disclosure rates between expert and non-expert mothers living with HIV in rural Nigeria. Given their experiential background, training, and empowerment, expert mothers are expected to have significantly higher rates of disclosure, and knowledge of their male partners’ HIV status compared to other WLHIV. However, this was not the case in our study findings; only male partner disclosure rates were significantly higher for expert versus non-expert mothers. There were no significant differences in disclosure to anyone and in knowledge of male partners’ HIV status. Among pregnant and postpartum women in sub-Saharan Africa, the pooled estimate of disclosure to anyone is 67%, and 63.9% disclosure to male partners is 64% [21]. All facilities surveyed in the study setting had, for up to 4 years prior to the study, provided peer support to WLHIV. It is thus reasonable to expect that non-expert study participants, who were all enrolled in care at these facilities, would have been in contact with a healthcare worker and/or peer counsellor who would have encouraged them to disclose, resulting in a high and comparable background rate of self-reported disclosure (>80%) among expert and non-expert mothers. We opine however, that the expectation of universal disclosure among the expert women, who serve as counsellors and role models, would also be reasonable.

Among our study cohort, non-expert mothers were more likely to have more education than expert mothers, and expert mothers were more likely to be married compared to non-expert mothers. It is possible that women with less education (and presumably less well-employed) may be more likely to seek opportunities as peer counsellors or join support groups to educate and/or empower themselves. Our study was not designed to determine if and how education and marital status had any impact on the differences in disclosure rates (if any) between the two groups. The available data is mixed on this issue: while Sarko et al. [13] reported that male partner disclosure was positively associated with facility-based delivery (important for PMTCT) among WLHIV in North-Central Nigeria, they did not find any significant associations between disclosure and education. Our study team’s findings from a different cohort of women in the same setting showed that disclosure status did not correlate with facility delivery [29]. In Uganda, Ngonzi et al reported that primary education was associated with a 3.5-fold increase in the odds of disclosure compared to those with post-primary education among pregnant WLHIV [30]. Another Ugandan study did find that having secondary education or higher, and being married or cohabiting were predictors of higher disclosure rates to male partners among women (HIV- positive and HIV-negative) who attended ANC [31].

Knowledge of partner HIV status—and in the case of polygamous unions, additional knowledge of co-wives’ status—is critical in the prevention of sexual transmission of HIV among couples, and PMTCT [32]. While we found no differences in knowledge of partner HIV status between expert and non-expert women, this knowledge was not universal. This finding is particularly important in our study setting, as our cohort of WLHIV was relatively young, mostly married, and expected to have more children. In addition, polygamous unions are common in Nigeria, even though polygamy was not highly prevalent among our study cohort. According to the 2018 Nigeria Demographic and Health Survey, 31% of currently married Nigerian women were in a polygamous union [33]. Thus, HIV-exposed children, uninfected partners, and co-wives, would be at risk of HIV infection if complete disclosure is not achieved within these families.

Further complicating the impact of peer support in PMTCT is poor expert mother-client disclosure. While expert mothers have been widely adopted in PMTCT programs, relatively little is said about consistent HIV status disclosure from expert mothers to clients. There is currently very limited data on non-disclosure among expert mothers with regard to male partners and clients. Hardon et al [34] in a four-country study, reported that HIV-status disclosure was negatively associated with membership in a support group. Some members considered their support group membership as a type of disclosure, perhaps negating the need for further disclosure to others who are not themselves HIV-positive, such as male partners and family members, for fear of stigma. Our study found that even among empowered role models such as expert mothers, HIV disclosure for prevention among other women (co-wives and clients) and for PMTCT did not consistently occur. This is presumably due to the persistently strong community-level stigma and discrimination that affect both expert and non-expert mothers. Ultimately, the inconsistency of disclosure by expert mothers to clients and male partners is not exemplary for other WLHIV, some of whom may subsequently work as peer counsellors.

To function effectively as role models and impactful change agents, expert mothers need to be able to reach out to the larger community to break down community-level barriers and stigma. As such, some PMTCT programs expect expert mothers to be willing and able to publicly disclose their HIV status as a pre-requisite for engagement [8]. To achieve this on a wider scale will necessitate strengthening expert mothers’ skills for public speaking, HIV status disclosure, and community sensitization and mobilization. In this process, major barriers to personal disclosure to male partners, co-wives (where applicable), and clients among expert mothers should be addressed directly. Our study and other reports show that expert mothers’ experiences and fear of stigma, discrimination and other negative consequences of disclosure mirror those of their clients [35]. This indicates that any strategies to improve disclosure rates among non-expert mothers should also target expert mothers.

### Study limitations

We did not conduct FGDs among non-expert WLHIV, and as such may have missed some findings to further explain the quantitative results. However, data from non-expert women in this study were intended for quantitative comparison only. We were unable to ascertain how many, and to what extent non-expert mothers had received formal/informal counselling and support for disclosure. In addition to social desirability bias, this limitation may have contributed to their high disclosure rates relative to WLHIV in general and expert mothers in particular. There were some missing data/non-responses for our quantitative data on disclosure status and knowledge of male partner status; which may have skewed our results.

### Conclusions

Any progress that PMTCT programs in Nigeria and similar settings have made in reducing stigma and improving disclosure will be limited, if empowered expert mothers are not outperforming less-experienced women. Persistent social, structural, and gendered barriers need intense grassroots-level community engagement and social-behavioral interventions to break the impasse. As PMTCT peer support interventions are scaled up, disclosure status should be reviewed amongst expert mothers at engagement, and periodically thereafter. This will better facilitate the desired behavioral modifications that individual and group peer support interventions aim to achieve.

## Supporting information

S1 File Survey questionnaire. (PDF)

S2 File Focus group discussion guide. (PDF)

S3 File Consolidated criteria for reporting qualitative studies (COREQ) checklist. (DOCX)
