# Supplementary material for: A mixed-methods assessment of disclosure of HIV status among expert mothers living with HIV in rural Nigeria
Source: PLoS One. 2020 Apr 30;15(4):e0232423. doi: 10.1371/journal.pone.0232423 (PMC7192376; doi:10.1371/journal.pone.0232423)
Supplement: S1 File — (PDF) [file pone.0232423.s001.pdf]

MoMent/Fogarty Study: Survey Questionnaire for Expert (Mentor Mother and Mother-to-Mother Support Group) and Non-Expert Women Living with HIV

Participant Study ID: ______________________________ Date:____________________

	I. Socio-Demographics (All)	Choose or Indicate Response
1	Can you please tell us how old you are?	a. <21<br>b. 21-30<br>c. 31 to 40<br>d. 41+ years
2	Are you currently in school or pursuing any studies?	Yes or No
3	What is the highest level you reached or have reached in school?	a. None<br>b. Primary<br>c. Secondary<br>d. Tertiary<br>e. Post-tertiary
4	Can you please tell us what work you do?	
5	**For Mentor Mothers (MM):** Aside from your work as an MM, what other work do you do, if any?	
6a	**For Mentor Mothers:** how long have you worked as an MM?	
6b	**For m2m support group members:** How long have you been a member of your support group?	
7	How many living children do you have?	
8	Can you share with us if you are currently pregnant?	
9	How old is your youngest child/baby now?	
10	Are you married, or do you have a male partner/boyfriend?	a. Single<br>b. Married<br>c. Divorced<br>d. Widowed<br>e. Separated
11	Do you have a religion you follow? If so, what is it?	a. Islam<br>b. Christianity<br>c. Other<br>d. None
12	When did you first learn that you had HIV?	
13	Are you on HIV treatment? If so, how long have you been on treatment?	
	**II. Disclosure (All)**	
1	Is there anyone close to you who knows about your HIV status?	
2	Have you told your husband/male partner your HIV status?	Yes or No or N/A
3	Does your husband/partner know your HIV status?	Yes, No, Not Sure, N/A
4a	Do you know your husband/partner's status?	Yes, No, or N/A
4b	If yes, what is his status?	Positive or Negative
5	Do you have other family (parents, siblings, other), whom you have told your HIV status?	• Parent(s) (mother and/or father)<br>• Sibling(s) (brother(s) and/or sister(s)<br>• Other
6	Are there others ie friends whom you have told your HIV status?	
7	Among the people you have disclosed to, who did you disclose to, first?	
	Thank you for participating in this interview. We greatly appreciate your time.	
