# Supplementary material for: A mixed-methods assessment of disclosure of HIV status among expert mothers living with HIV in rural Nigeria
Source: PLoS One. 2020 Apr 30;15(4):e0232423. doi: 10.1371/journal.pone.0232423 (PMC7192376; doi:10.1371/journal.pone.0232423)
Supplement: S2 File — (PDF) [file pone.0232423.s002.pdf]

INSPIRE MoMent Study FGD Guide: Mentor Mothers and m2m Support groups

(*After ice-breaker introduction):* If there are no further questions, we would like to begin the discussion. *(Begin recording).*

MAIN DISCUSSION

The initial question:

Let's talk about pregnant women and their care in the clinic.

1. What comes to mind when you think about the care pregnant women receive in the clinics in this area? What are the good things about coming to clinic? What are the bad things?
   What has been your experience, or that of anyone that you know?
   Probe: Fear of/apprehension with going to the community clinic was mentioned a lot. Tell me a bit more about that.
   Probe: if some of the group members talk about delivery at home ask: Tell us about how/why you would deliver at home/with the traditional birth attendant?

2. What about if the woman has HIV? Are there any differences in the care HIV+ women get from clinics?

A. ANTENATAL CARE (ANC) SERVICES

We will now talk about ANC/PMTCT services provided, and how happy or not happy we are with them.

3. First, tell us a bit about how you travel to the clinics and the challenges you face in getting here. Probe: Are the services too far from where you live or hard to get to?

4. Next, tell us about how you felt you were treated when you came into the clinic to get medical care. What usually happens- are you seen right away or do you have to wait long?
   Probe: why is that the case?
   Probe: Do you feel that the staff treated you with respect and value your point of view?

5. Is the staff usually ready to attend to you for the tests or the medicine you need?
   Probe; If not what do you think we should do to improve this?

6. Do you have any special religious or cultural requirements- such as needing to be seen only by the same gender? Probe: How has the staff responded to these needs? Overall have you found the staff to be welcoming?

7. Have you found any fault with the government or local politicians/chiefs that have made it challenging to visit the clinic or to get the care you need? How can we/they solve these problems?

B. COMMUNICATION – ISSUES RELATED TO LANGUAGE, BEING TALKED WITH AND HEARD.

The next discussion focuses on the degree to which you feel that the issues you are raising are understood by the program staff.

1. Tell us what happens when our doctors, nurses and other staff members talk to you about being HIV positive?
2. Do you feel they understand what you were trying to tell them?
   Probes: Did they speak the same language? Did you feel that you were being ignored or brushed aside when you attempted to speak?

C. DISCUSSION OF STRESS/STIGMA – AN EXPLORATION OF THE ISSUES OF STRESS AND STIGMA RELATING TO LIVING WITH HIV/AIDS

The next discussion is focused on the perceptions of stress and stigma related to living with HIV/AIDS.

1. Tell us about the types of stress women living with HIV are facing in coping with HIV/AIDS.
2. How do you feel about taking your medications? How about disclosing/sharing your HIV status with your peer clients, partner, family or friends?
3. Tell us about the ways in which you may feel/or felt isolated and or stigmatized by others in your community.
   Probes. How often do you feel stressed? How sensitive were other people to your stress?

D. ACCEPTABILITY, ROLES AND IMPACT OF MENTOR MOTHERS (MM only)

The next part of the focus group looks at the types of (informal or formal) support mothers receive in the prevention of mother-to-child transmission (PMTCT) program. Let's talk about your work as Mentor Mothers (MM).

1. First, tell us a bit about how you became a Mentor Mother. What motivated you?
2. What exactly are you supposed to do for your clients? What do you talk to them about? What do you tell them about yourself?
3. What have your experiences as an MM been like? Do you think you are making/have made a difference?
4. How do your clients feel about having a helper or support person to talk to them? Probe- Have clients been open to having a helper support them? If so what are the things they think you have been most helpful with?
5. Have clients been open to have you visiting them at home? What are they worried about, if they don't want you to visit at home?
6. How can the MMs/the MM program be more helpful to PMTCT clients?

7. How has being an MM affected your family life? Probe: How does your husband/partner feel about you working as a MM?
8. Do you have another job? How has that been affected?

E. ACCEPTABILITY AND EFFICACY OF INFORMAL SUPPORT PROVIDED BY M2M MEMBERS TO EACH OTHER AND TO NON-MEMBERS (M2M only)

The next part of the focus group looks at M2M support groups. Let's talk about your involvement in support groups with other mothers living with HIV.

1. First, tell us a bit about how you learned/found out about the M2M support groups.
2. Have you personally benefited from being a member? Probe: Has your involvement in this group been helpful for you and/or your family? If yes, how so? If no, why not? Has your membership of this support group made a difference in your ability to share your HIV status with others?
3. Has your involvement in this group allowed you to help non-members? If so, how have you been able to help others? Have others you have tried to help, been accepting of your advice or help?
4. How can we engage women living with HIV or use support groups to reach more mothers like you?

Closing

We would like to finish our discussion by asking if there are any suggestions you have for us or anything you would like to say in closing.

We would like to thank you for spending time with us, we appreciate all that you told us and look forward to using this information to help improve PMTCT services at clinics in this community and in Nigeria.

(End recording.)
