# Supplementary material for: A mixed-methods assessment of disclosure of HIV status among expert mothers living with HIV in rural Nigeria
Source: PLoS One. 2020 Apr 30;15(4):e0232423. doi: 10.1371/journal.pone.0232423 (PMC7192376; doi:10.1371/journal.pone.0232423)
Supplement: S3 File — (DOCX) [file pone.0232423.s003.docx]

**Consolidated criteria for reporting qualitative studies (COREQ) checklist for:**

**“A Mixed-Methods Assessment of** **Disclosure of HIV Status among Expert Mothers Living with HIV in Rural Nigeria.”**

| **No.** | **Item** | **Guide Questions/Description** | **Response (and Location in Manuscript where applicable)** |
| --- | --- | --- | --- |
| **Domain 1: Research Team and Reflexivity** | | | |
| Personal Characteristics | | | |
| 1 | Interviewer/facilitator | Which author/s conducted the interview or focus group? | SE, LJC and NASA conducted the focus groups. |
| 2 | Credentials | What were the researcher’s credentials? E.g. PhD, MD | SE (MPH), LJC (PhD), NASA (MD). |
| 3 | Occupation | What was their occupation at the time of the study? | SE, Research Associate  LJC, Social Science Professor  NASA, Technical Advisor Paediatric HIV Treatment and Prevention, and Principal Investigator. (Methods-Qualitative Data Collection). |
| 4 | Gender | Was the researcher male or female? | Females: AO, NASA, SE. Males: CI, HRO, HES, LJC. Overall, data analysis team comprised 5 females and 4 males.  (Methods-Qualitative Data Collection). |
| 5 | Experience and training | What experience or training did the researcher have? | For those who facilitated the FGDs:  SE (Trained; plus 3 years of conducting and analysing qualitative studies and surveys in study setting)  LJC (over 25 years’ experience in teaching, conducting, analysing and publishing qualitative studies)  NASA (Trained; plus 4 years of conducting and analysing qualitative studies in study setting and over 10 years of research experience). |
| **Relationship with participants** | | | |
| 6 | Relationship established | Was a relationship established prior to study commencement? | No, there was no established relationship with participants prior to study commencement beyond recruitment activities. |
| 7 | Participant knowledge of the interviewer | What did the participants know about the researcher? e.g. personal goals, reasons for doing the research | During orientation to study and consent process, researchers introduced themselves, stating where they worked (with an NGO/university and not the health facility or government), and reasons for doing the research, namely, to improve the quality of health services to women living with HIV. |
| 8 | Interviewer characteristics | What characteristics were reported about the interviewer/facilitator? Eg Bias, assumptions, reasons and interests in the research topic | Characteristics of interviewers and objectives are presented above and/or in the manuscript. The PI of the study, NASA, is female, and a paediatrician interested in the prevention of HIV among children and in maternal survival and long-term health in resource-limited settings. The Social Scientist, LJC, is male and interested in the psychology and social determinants of health and disease among populations of African descent. SE, female, has interest in the improvement of maternal and child health in Nigeria. |
| **Domain 2: Study Design** | | | |
| Theoretical Framework | | | |
| 9 | Methodological orientation and Theory | What methodological orientation was stated to underpin the study? e.g. grounded theory, discourse analysis, ethnography, phenomenology, content analysis | Grounded theory with constant comparative method content analysis (Methods-Qualitative Analysis). |
| Participant selection | | | |
| 10 | Sampling | How were participants selected? e.g. purposive, convenience, consecutive, snowball | Purposive sampling for all participants. (Methods-Participant Recruitment). |
| 11 | Method of approach | How were participants approached? e.g. face-to-face, telephone, mail, email | First by healthcare workers (through phone calls and in person) to ascertain interest, then those interested were approached by research team in person. |
| 12 | Sample size | How many participants were in the study? | A total of 137 participants (37 for 4 FGDs and surveys; and 100 for surveys) (Methods and Results). |
| 13 | Non-participation | How many people refused to participate or dropped out? Reasons? | 3 expert mothers declined to participate in FGDs; 1 due to scheduling conflict, 2 unknown reasons-did not show. |
| **Setting** | | | |
| 14 | Setting of data collection | Where was the data collected? e.g. home, clinic, workplace | There were 16 primary healthcare facilities and 4 secondary facilities involved, all located in rural areas. Data were collected in private rooms of these facilities during non-clinic hours. (Methods-Study Setting). |
| 15 | Presence of non-participants | Was anyone else present besides the participants and researchers? | No one else was present besides participants and researchers. (Methods-Participant Recruitment and Data Collection) . |
| 16 | Description of sample | What are the important characteristics of the sample? e.g. demographic data, date | There were two groups of participants, who were all women living with HIV: 1. Expert mothers (mentor mothers and experienced mother-to-mother support group members) participated in FGDs and surveys 2. Non-expert mothers (women with little or no experience with support groups and no history of working as mentor mothers) participated only in surveys. (Methods and Results). |
| **Data collection** | | | |
| 17 | Interview guide | Were questions, prompts, guides provided by the authors? Was it pilot tested? | FGD guides and survey questionnaires used for the study are provided (Supporting Information). FGD guides were adjusted as focus groups were conducted, per emerging data and recurring questions. Survey guides were pilot-tested among 10 women before finalization and implementation among study participants. |
| 18 | Repeat interviews | Were repeat interviews carried out? If yes, how many? | No, repeat interviews of participants were not conducted. |
| 19 | Audio/visual recording | Did the research use audio or visual recording to collect the data? | FGDs were audio-recorded, and later transcribed. There were no visual recordings. (Methods). |
| 20 | Field notes | Were field notes made during and/or after the interview or focus group? | Yes, field notes were made during the FGDs to augment data analysis and interpretation. (Methods-Qualitative Data Collection). |
| 21 | Duration | What was the duration of the interviews or focus group? | Each FGD took 1 ½ to 2 hours long. (Methods-Qualitative Data Collection). |
| 22 | Data saturation | Was data saturation discussed? | Multiple FGDs were conducted among all eligible and available expert mothers in our catchment area, and thus we were confident that we achieved saturation in terms of sample adequacy-being able to interview the majority of the target expert mother sample. (Methods-Qualitative Analysis). |
| 23 | Transcripts returned | Were transcripts returned to participants for comment and/or correction? | No. Nearly 50% of participants in this rural setting could either not read or write or were not literate enough to be able to read the transcripts regardless of whether they were in English or local language. A verbal member check was not performed either, however the diverse and context-experienced facilitator and analyst team and our iterative analysis approach provided robust interpretation (Methods-Qualitative Analysis). |
| **Domain 3: Analysis and Findings** | | | |
| **Data analysis** | | | |
| 24 | Number of data coders | How many data coders coded the data? | Overall analysis was manually performed by an interactive panel of eight paired researchers, which included FGD facilitators. The PI (NASA) and Social Scientist (LJC) provided the coding list, and with an additional 6 research staff coded the data, including SE. AO (DrPH, MPhil Sociology & Development studies) additionally performed independent analysis (Methods-Qualitative Data Analysis). |
| 25 | Description of the coding tree | Did authors provide a description of the coding tree? | The root pre-set code words were “disclosure”, “stigma”, “discrimination”, and “support.” The pre-set codes served as the root of the coding trees for the analysis. The process was supplemented by emergent coding using the constant comparative approach. (Methods-Qualitative Data Analysis). |
| 26 | Derivation of themes | Were themes identified in advance or derived from the data? | Both-even though we pre-coded some of the codes, we used the grounded theory approach to derive specific themes from the data. (Methods-Qualitative Data Analysis). |
| 27 | Software | What software, if applicable, was used to manage the data? | No specialized qualitative software was used to manage the data. |
| 28 | Participant checking | Did participants provide feedback on the findings? | No. Member checks were not performed. |
| **Reporting** | | | |
| 29 | Quotations presented | Were participant quotations presented to illustrate the themes / findings? Was each quotation identified? e.g. participant number | Participant quotations are presented and identified by participant category and number: mentor mother or support group member. (Throughout Results section). |
| 30 | Data and findings consistent | Was there consistency between the data presented and the findings? | Yes, Discussion of findings was written to align with Results/data presented. |
| 31 | Clarity of major themes | Were major themes clearly presented in the findings? | Yes, disclosure to male partners and family members as well as knowledge of male partner HIV status are presented in both quantitative and qualitative results. |
| 32 | Clarity of minor themes | Is there a description of diverse cases or discussion of minor themes? | Yes, Expert Mother disclosure to peers, and disclosure to co-wives in polygamous unions emerged as minor themes that were described (Results). |
